# Supplementary material for: Is therapeutic inertia present in hyperglycaemia, hypertension and hypercholesterolaemia management among adults with type 2 diabetes in three health clinics in Malaysia? a retrospective cohort study
Source: BMC Fam Pract. 2021 Jun 11;22:111. doi: 10.1186/s12875-021-01472-2 (PMC8194183; doi:10.1186/s12875-021-01472-2)
Supplement: Supplementary file 1 — Additional file 1: Table S1. Characteristics of patients and uncontrolled HbA1c according to the anti-diabetics therapeutic changes in 2015, n (row %) unless stated otherwise. Table S2. Characteristics of patients and at least two consecutive blood pressure not at targets according to the anti-hypertensive therapeutic changes in 2015, n (row %) unless stated otherwise. Table S3. Characteristics of patients and uncontrolled low density lipoprotein-cholesterol according to the lipid lowering therapeutic changes in 2015, n (row %) unless stated otherwise. [file 12875_2021_1472_MOESM1_ESM.docx]

Is therapeutic inertia present in hyperglycaemia, hypertension and hypercholesterolaemia management among adults with type 2 diabetes in three health clinics in Malaysia? A retrospective cohort study

Boon-How Chew^1,*^, Husni Hussain^2^, Ziti Akthar Supian^3^

^1^Department of Family Medicine, Faculty of Medicine and Health Sciences, Universiti Putra Malaysia, UPM Serdang, Selangor, Malaysia.

^2^Salak Health Clinic, Jalan Salak, 43900 Sepang Selangor, Malaysia.

^3^Seri Kembangan Health Clinic, Jalan Besar, Taman Muhibbah, 43300 Seri Kembangan, Selangor, Malaysia.

*** Correspondence:**Boon-How Chew, Department of Family Medicine, Faculty of Medicine & Health Sciences, Universiti Putra Malaysia, 43400 Serdang, Selangor, Malaysia.

Email: [chewboonhow@upm.edu.my](mailto:chewboonhow@upm.edu.my)

**Table S1** Characteristics of patients and uncontrolled HbA1c according to the anti-diabetics therapeutic changes in 2015, n (row %) unless stated otherwise

|  | Total (column %) | Anti-diabetic therapeutic changes | | | χ^2^/F/KW value | P value |
| --- | --- | --- | --- | --- | --- | --- |
|  |  | No change | Stepping  up | Stepping down |  |  |
| HbA1c ≥ 7.0%  HbA1c < 7.0% | Oral anti-diabetic agents | | | | | |
|  | 367 (73.7)  131 (26.3) | 236 (64.3)  89 (67.9) | 74 (20.2)  30 (22.9) | 57 (15.5)  12 (9.2) | 3.37 | 0.185 |
| HbA1c ≥ 7.0%  HbA1c < 7.0% | Insulin | | | | | |
|  | 365 (73.7)  130 (26.3) | 170 (46.6)  118 (90.8) | 167 (45.8)  9 (6.9) | 28 (7.7)  3 (2.3) | 77.23 | <0.001 |
| Clinic  Seri Kembangan  Dengkil  Salak | 189 (34.3)  106 (19.2)  256 (46.5) | 113 (59.8)  57 (53.8)  155 (60.5) | 71 (37.6)  43 (40.6)  77 (30.1) | 5 (2.6)  6 (5.7)  24 (9.4) | 11.54 | 0.021 |
| Age in year, mean (SD) n= 551 | 60 (10.0) | 61 (10.2) | 59 (9.5) | 59 (9.9) | 2.15 | 0.117 |
| Gender  Female | 293 (53.2) | 183 (62.5) | 92 (31.4) | 18 (6.1) | 3.25 | 0.197 |
| Male | 258 (46.8) | 142 (55.0) | 99 (38.4) | 17 (6.6) |  |  |
| Ethnicity  Malay | 270 (49.5) | 164 (60.7) | 84 (31.1) | 22 (8.1) | 7.64 | 0.106 |
| Chinese | 138 (25.3) | 85 (61.6) | 46 (33.3) | 7 (5.1) |  |  |
| Indian | 137 (25.1) | 72 (52.6) | 59 (43.1) | 6 (4.4) |  |  |
| Marital status  Married/living with a partner | 431 (82.3) | 249 (57.8) | 156 (36.2) | 26 (6.0) | 1.81 | 0.404 |
| Divorced/Separated/Widow/Single | 93 (17.7) | 59 (63.4) | 27 (29.0) | 7 (7.5) |  |  |
| Education level  Primary/None | 218 (40.3) | 126 (57.8) | 80 (36.7) | 12 (5.5) | 1.21 | 0.876 |
| Secondary | 240 (44.4) | 144 (60.0) | 79 (32.9) | 17 (7.1) |  |  |
| Tertiary | 83 (15.3) | 50 (60.2) | 27 (32.5) | 6 (7.2) |  |  |
| Employment status  Employed | 246 (44.8) | 140 (60.0) | 91 (37.0) | 15 (6.1) | 4.81 | 0.308 |
| Retired | 137 (25.0) | 76 (55.5) | 49 (35.8) | 12 (8.8) |  |  |
| Unemployed/Homemaker | 166 (30.2) | 108 (65.1) | 50 (30.1) | 8 (4.8) |  |  |
| Life event in the past 6 months  Yes | 51 (16.6) | 35 (68.6) | 14 (27.5) | 2 (3.9) | 2.01 | 0.366 |
| No | 257 (83.4) | 149 (58.0) | 95 (37.0) | 13 (5.1) |  |  |
| Any diabetes complication  No | 484 (87.8) | 292 (60.3) | 163 (33.7) | 29 (6.0) | 3.16 | 0.206 |
| Yes | 67 (12.2) | 33 (49.3) | 28 (41.8) | 6 (9.0) |  |  |
| Total number of prescribed medication, mean (SD) n= 549 | 5 (1.8) | 5 (1.8) | 6 (1.8) | 6 (2.0) | 11.60 | < 0.001 |
| Diabetes Duration in year, median (IQR) n= 539 | 5 (6.0) | 4 (5.0) | 6 (6.0) | 7 (8.0) | 31.56 | < 0.001 |
| HPT Duration in year, median (IQR) n= 406 | 5 (7.0) | 5 (7.0) | 6 (7.0) | 5.5 (5.0) | 0.95 | 0.622 |
| BMI, mean (SD) n= 541 | 29 (5.4) | 29 (5.5) | 29 (5.2) | 29 (5.3) | 0.40 | 0.669 |
| Hypertension status  No | 117 (21.5) | 61 (52.1) | 45 (38.5) | 11 (9.4) | 3.83 | 0.148 |
| Yes | 426 (78.5) | 259 (60.8) | 143 (33.6) | 24 (5.6) |  |  |
| Dyslipidaemia status  No | 300 (56.6) | 181 (60.3) | 94 (31.3) | 25 (8.3) | 6.20 | 0.045 |
| Yes | 230 (43.4) | 132 (57.4) | 89 (38.7) | 9 (3.9) |  |  |

Stepping up included dose increment or/and replacement with a stronger medication; stepping down is the other ways round; F tests are adjusted for all pairwise comparisons within a row using the Bonferroni correction; KW= Kruskal-Wallis test; IQR= interquartile range; BMI= body mass index; HPT= hypertension; SD= standard deviation

**Table S2** Characteristics of patients and at least two consecutive blood pressure not at targets according to the anti-hypertensive therapeutic changes in 2015, n (row %) unless stated otherwise

|  | Total (column %) | AHA Therapeutic changes | | | χ^2^/F/KW value | P value |
| --- | --- | --- | --- | --- | --- | --- |
|  |  | No change | Stepping  up | Stepping down |  |  |
| At least 2 consecutive BP ≥ 140/90 mmHg  In the whole 2015  Yes | 218 (39.9) | 111 (50.9) | 75 (34.4) | 32 (14.7) | 48.77 | < 0.001 |
| No | 328 (60.1) | 251 (76.5) | 36 (11.0) | 41 (12.5) |  |  |
| In the first half of the year 2015  Yes | 148 (27.1) | 72 (48.6) | 53 (35.8) | 23 (15.5) | 34.19 | < 0.001 |
| No | 398 (72.9) | 290 (72.9) | 58 (14.6) | 50 (12.6) |  |  |
| In the second half of the year 2015  Yes | 107 (21.1) | 48 (44.9) | 40 (37.4) | 19 (17.8) | 27.91 | < 0.001 |
| No | 399 (78.9) | 284 (71.2) | 67 (16.8) | 48 (12.0) |  |  |
| Seri Kembangan Health Clinic  Dengkil Health Clinic  Salak Health Clinic | 190 (34.2)  107 (19.2)  259 (46.6) | 104 (54.7)  89 (83.2)  176 (68.0) | 64 (33.7)  11 (10.3)  38 (14.7) | 22 (11.6)  7 (6.5)  45 (17.4) | 41.92 | < 0.001 |
| Age in year, mean (SD) n= 556 | 60.2 (9.98) | 58.8 (10.00) | 62.9 (9.16) | 62.8 (9.80) | 10.74 | < 0.001 |
| Female | 294 (52.9) | 200 (68.0) | 52 (17.7) | 42 (14.3) | 2.84 | 0.242 |
| Male | 262 (47.1) | 169 (64.5) | 61 (23.3) | 32 (12.2) |  |  |
| Malay | 273 (49.6) | 184 (67.4) | 45 (16.5) | 44 (16.1) | 20.24 | < 0.001 |
| Chinese | 138 (25.1) | 77 (55.8) | 45 (32.6) | 16 (11.6) |  |  |
| Indian | 139 (25.3) | 103 (74.1) | 23 (16.5) | 13 (9.4) |  |  |
| Marital status  Married/living with a partner | 435 (82.2) | 291 (66.9) | 85 (19.5) | 59 (13.6) | 0.42 | 0.812 |
| Divorced/Separated/Widow/Single | 94 (17.8) | 60 (63.8) | 21 (22.3) | 13 (13.8) |  |  |
| Education level  Primary/None | 219 (40.1) | 144 (65.8) | 49 (22.4) | 26 (11.9) | 2.73 | 0.604 |
| Secondary | 243 (44.5) | 168 (69.1) | 43 (17.7) | 32 (13.2) |  |  |
| Tertiary | 84 (15.4) | 53 (63.1) | 17 (20.2) | 14 (16.7) |  |  |
| Employed | 248 (44.8) | 180 (72.6) | 39 (15.7) | 29 (11.7) | 12.64 | 0.013 |
| Retired | 140 (25.3) | 79 (56.4) | 41 (29.3) | 20 (14.3) |  |  |
| Unemployed/Homemaker | 166 (30.0) | 108 (65.1) | 33 (19.9) | 25 (15.1) |  |  |
| Life event in the past 6 months  Yes | 53 (17.0) | 31 (58.5) | 14 (26.4) | 8 (15.1) | 2.52 | 0.284 |
| No | 259 (83.0) | 178 (68.7) | 57 (22.0) | 24 (9.3) |  |  |
| Any diabetes complication  No | 488 (87.8) | 329 (67.4) | 99 (20.3) | 60 (12.3) | 3.75 | 0.153 |
| Yes | 68 (12.2) | 40 (58.8) | 14 (20.6) | 14 (20.6) |  |  |
| Total number of prescribed medication, mean (SD) n= 554 | 5 (1.8) | 5 (1.8) | 5 (1.7) | 6 (2.1) | 4.88 | 0.008 |
| Diabetes Duration in year, mean (SD) n= 544 | 5 (6.0) | 4 (5.0) | 6 (7.0) | 5 (7.0) | 5.58 | 0.061 |
| HPT Duration in year, mean (SD) n= 409 | 5 (7.0) | 5 (6.0) | 6 (8.0) | 7 (10.0) | 5.99 | 0.050 |
| BMI, mean (SD) n= 546 | 29 (5.4) | 29 (5.3) | 28 (5.7) | 30 (5.3) | 3.01 | 0.050 |
| Hypertension status  No | 119 (21.7) | 93 (78.2) | 19 (16.0) | 7 (5.9) | 10.84 | 0.004 |
| Yes | 429 (78.3) | 271 (63.2) | 92 (21.4) | 66 (15.4) |  |  |
| Dyslipidaemia status  No | 304 (56.8) | 204 (67.1) | 53 (17.4) | 47 (15.5) | 3.83 | 0.148 |
| Yes | 231 (43.2) | 154 (66.7) | 52 (22.5) | 25 (10.8) |  |  |

Stepping up included dose increment or/and replacement with a stronger medication; stepping down is the other ways round; F tests are adjusted for all pairwise comparisons within a row using the Bonferroni correction; KW= Kruskal-Wallis test; IQR= interquartile range

AHA= antihypertensive agents BMI= body mass index; HPT= hypertension; SD= standard deviation

**Table S3** Characteristics of patients and uncontrolled low density lipoprotein-cholesterol according to the lipid lowering therapeutic changes in 2015, n (row %) unless stated otherwise

|  | Total (column %) | LLA Therapeutic changes | | | χ^2^/F/ KW value | P value |
| --- | --- | --- | --- | --- | --- | --- |
|  |  | No change | Stepping  up | Stepping down |  |  |
| LDL ≥ 2.6 mmol/L  Yes  No | 206 (58.0)  149 (42.0) | 126 (61.2)  122 (81.9) | 59 (28.6)  17 (11.4) | 21 (10.2)  10 (6.7) | 18.50 | <0.001 |
| Clinic  Seri Kembangan  Dengkil  Salak | 190 (34.2)  107 (19.2)  259 (46.6) | 144 (75.8)  86 (80.4)  170 (65.6) | 24 (12.6)  19 (17.8)  61 (23.6) | 22 (11.6)  2 (1.9)  28 (10.8) | 17.94 | 0.001 |
| Age in year, mean (SD) n= 556 | 60 (10.0) | 61 (10.3) | 59 (8.8) | 59 (9.6) | 2.13 | 0.120 |
| Gender  Female | 294 (52.9) | 209 (71.1) | 58 (19.7) | 27 (9.2) | 0.43 | 0.806 |
| Male | 262 (47.1) | 191 (72.9) | 46 (17.6) | 25 (9.5) |  |  |
| Ethnicity  Malay | 273 (49.6) | 189 (69.2) | 55 (21.1) | 29 (10.6) | 4.97 | 0.290 |
| Chinese | 138 (25.1) | 103 (74.6) | 20 (14.5) | 15 (10.9) |  |  |
| Indian | 139 (25.3) | 103 (74.1) | 28 (20.1) | 8 (5.8) |  |  |
| Marital status  Married/living with a partner | 435 (82.2) | 305 (70.1) | 85 (19.5) | 45 (10.3) | 4.15 | 0.126 |
| Divorced/Separated/Widow/Single | 94 (17.8) | 74 (78.7) | 16 (17.0) | 4 (4.3) |  |  |
| Education level  Primary/None | 219 (40.1) | 166 (68.7) | 35 (16.0) | 18 (8.2) | 3.30 | 0.509 |
| Secondary | 243 (44.5) | 167 (68.7) | 51 (21.0) | 25 (10.3) |  |  |
| Tertiary | 84 (15.4) | 62 (73.8) | 16 (19.0) | 6 (7.1) |  |  |
| Employment status  Employed | 248 (44.8) | 173 (69.8) | 49 (19.8) | 26 (10.5) | 3.87 | 0.423 |
| Retired | 140 (25.3) | 110 (78.6) | 20 (14.3) | 10 (7.1) |  |  |
| Unemployed/Homemaker | 166 (30.0) | 117 (70.5) | 33 (19.9) | 16 (9.6) |  |  |
| Life event in the past 6 months  Yes | 53 (17.0) | 39 (73.6) | 8 (15.1) | 6 (11.3) | 0.37 | 0.832 |
| No | 259 (83.0) | 187 (72.2) | 47 (18.1) | 25 (9.7) |  |  |
| Any diabetes complication  No | 488 (87.8) | 348 (71.3) | 95 (19.5) | 45 (9.2) | 1.53 | 0.465 |
| Yes | 68 (12.2) | 52 (76.5) | 9 (13.2) | 7 (10.3) |  |  |
| Total number of prescribed medication, mean (SD) n= 554 | 5 (1.8) | 5 (1.8) | 5 (1.8) | 5 (1.9) | 2.47 | 0.085 |
| Diabetes Duration in year, median (IQR) n= 544 | 5 (6.0) | 5 (6.0) | 4 (6.0) | 4 (4.0) | 4.80 | 0.091 |
| HPT Duration in year, median (IQR) n= 409 | 5 (7.0) | 5 (7.0) | 5 (8.0) | 4 (8.0) | 1.46 | 0.482 |
| BMI, mean (SD) n= 546 | 29 (5.4) | 28 (5.5) | 29 (4.4) | 30 (5.7) | 1.83 | 0.162 |
| Hypertension status  No | 119 (21.7) | 79 (66.4) | 32 (26.9) | 8 (6.7) | 7.46 | 0.024 |
| Yes | 429 (78.3) | 315 (73.4) | 70 (16.3) | 44 (10.3) |  |  |
| Dyslipidaemia status  No | 304 (56.8) | 211 (69.4) | 63 (20.7) | 30 (9.9) | 1.20 | 0.549 |
| Yes | 231 (43.2) | 170 (73.6) | 40 (17.3) | 21 (9.1) |  |  |

Stepping up included dose increment or/and replacement with a stronger medication; stepping down is the other ways round; F tests are adjusted for all pairwise comparisons within a row using the Bonferroni correction; KW= Kruskal-Wallis test; IQR= interquartile range

BMI= body mass index; HPT= hypertension; LLA= lipid lowering agents; SD= standard deviation
